# Virus-Negative Active Lymphocytic Myocarditis Progressing to a Fibrotic Stage

**DOI:** 10.1155/2011/740928

**Published:** 2011-03-16

**Authors:** Edouard Gerbaud, Anne Vital, Matthew Erickson, Michel Montaudon, Emmanuel Harcaut, Jean luc Pellegrin, François Laurent, Pierre Coste

**Affiliations:** ^1^Soins Intensifs Cardiologiques—Plateau de Cardiologie Interventionnelle, CHU de Bordeaux, 5 Avenue de Magellan, 33604 Pessac, France; ^2^Laboratoire d'Anatomie Pathologique, Hôpital Pellegrin, Place Amélie Raba Léon, 33076 Bordeaux, France; ^3^Unité d'Imagerie Thoracique et Cardiovasculaire, CHU de Bordeaux, 5 Avenue de Magellan, 33604 Pessac, France; ^4^Service de Médecine Interne et Maladies Infectieuses, CHU de Bordeaux, Groupe Hospitalier Sud, 33604 Pessac, France

## Abstract

We report a fairly special case of lymphocytic myocarditis progressing to a fibrotic stage, described using multimodality imaging and confirmed on histopathology. This paper presents an uncommon diagnosis with a probable guarded prognosis.

## 1. Introduction

Myocarditis is a disease characterised by a polymorphic clinical presentation and appears to be a major cause of sudden, unexpected death in adults aged <40 years [1]. A prominent lymphocytic infiltrate histologically characterizes the majority of biopsy-verified myocarditis. We report a fairly special case of an apparently lymphocytic myocarditis with reparative fibrosis, described using multimodality imaging and confirmed on histopathology. 

## 2. Case Presentation

**Figure 1:**
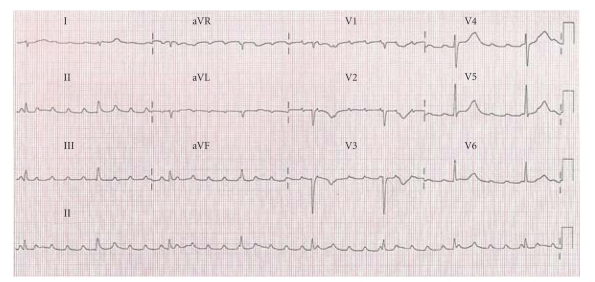
Twelve-lead ECG demonstrating a complete atrioventricular block.

**Figure 2:**
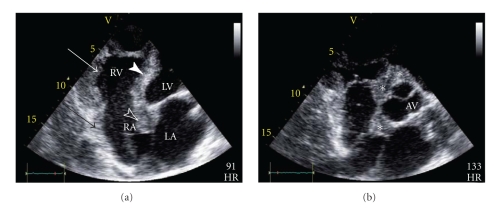
Apical two-dimensional four-chamber (a) echocardiogram showing marked thickening of the right ventricle (arrow), right atrium (empty arrow), interatrial septum (empty arrowhead), and basal to mid interventricular septum (arrowhead) associated with a pericardial effusion. Aortic valve short-axis view (b) demonstrating that the thickened tissue (asterisk) extends around the root of aorta. RA indicates right atrium; LA, left atrium; RV, right ventricle; LV, left ventricle; AV, aortic valve.

**Figure 3:**
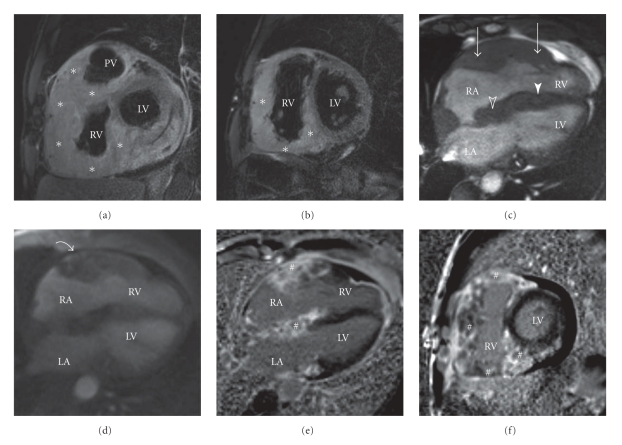
T2-weighted short inversion-time, inversion-recovery (STIR) breath hold pulse sequences ((a) and (b)) showing concentric thickening and oedema of the right ventricle. The infiltration (asterisk) extends around the root of the pulmonary artery. Four-chamber SSFP cine view (c) showing infiltration of the right ventricle (arrow), right atrium (empty arrow), interatrial septum (empty arrowhead), and basal to mid interventricular septum (arrowhead). Four-chamber first-pass T1-weighted multishot gradient-echo echo-planar sequence (d) shows partial hyperenhancement (arrow) of the right ventricle in support of an inflammatory process. Four-chamber (e) and short-axis (f) three-dimensional phase-sensitive inversion recovery sequences demonstrating widespread and heterogeneous enhancement (#) of the right atrium and ventricle. RA indicates right atrium; LA, left atrium; RV, right ventricle; LV, left ventricle; PV, pulmonary valve.

**Figure 4:**
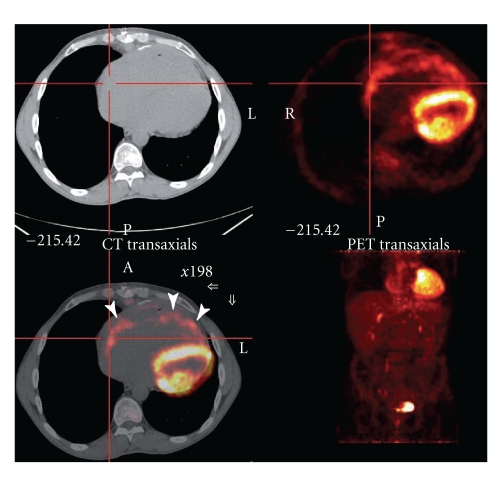
FDG-PET in the transaxial plane shows moderate FDG uptake (arrows) involving mainly the right heart.

A 49-year-old farm laborer was referred to our hospital with complete heart block, incidentally discovered when he presented with persistent left sciatica despite analgesic treatment. No personal or familial history except a smoking habit was reported. The sciatica did not require immediate surgical management according to the physicians' assessment after a spinal magnetic resonance imaging. Electrocardiogram (Figure 1) revealed complete atrioventricular block associated with atrial tachycardia. Thus, this patient was sent to our intensive care unit for cardiac investigation. Clinical history revealed New York Heart Association (NYHA) class II dyspnea, associated with clinical signs of global heart failure. Chest radiography demonstrated cardiomegaly and bilateral hilar overload. Transthoracic echocardiography (Figure 2(a), see Movie 1 Supplementary Material available online at doi:10.1155/2011/740928) revealed extensive localized thickening of the right ventricle, right atrium, interatrial septum, and basal to mid interventricular septum associated with a pericardial effusion. The infiltration extended around the root of the pulmonary artery and aorta (Figure 2(b), Movie 2). Left ventricular systolic function was slightly impaired (ejection fraction = 54%). Analysis of tissue Doppler indices showed elevated left ventricular filling pressures (E/E′ ratio = 16.4). Blood sample tests found an isolated inflammatory syndrome: C-Reactive Protein raised to 50 mg/L. Nt-proBNP increased to 1150 pg/mL (*N* < 125 pg/mL). Serological tests for a bacterial, fungal, or immunological (including anti-nuclear antibodies, antineutrophil cytoplasmic antibodies, rheumatoid factor) cause were negative. QuantiFERON-TB Gold test was negative, and the angiotensin-converting enzyme level was normal. Cardiovascular magnetic resonance (CMR) imaging was performed to localize and characterize the nature of the tissue thickening. Four-chamber (Figure 3(c), Movie 3), apical short-axis, and long-axis (Movie 4) cine images were performed using steady-state free precession (SSFP) cine sequences. The right ventricular wall was akinetic, with preservation of apical contractility. Short-axis dark-blood T2-weighted (Figures 3(a) and 3(b)) sequences confirmed concentric thickening and oedema of the right ventricle. First-pass perfusion (Figure 3(d), Movie 5) showed enhancement of the right ventricular and interventricular septum infiltration consistent with an active inflammatory process. Delayed enhancement CMR (Figures 3(e) and 3(f)) sequences demonstrated widespread and heterogeneous enhancement of the right ventricle. Combined (18)F-fluoro-2-deoxyglucose positron emission tomography (FDG PET)/computed tomography (CT) was performed to look for primary malignant lesion (Figure 4) showing moderate FDG uptake involving mainly the right heart chambers of the heart. This whole body exam did not detect any extracardiac locations of FDG uptake. 

**Figure 5:**
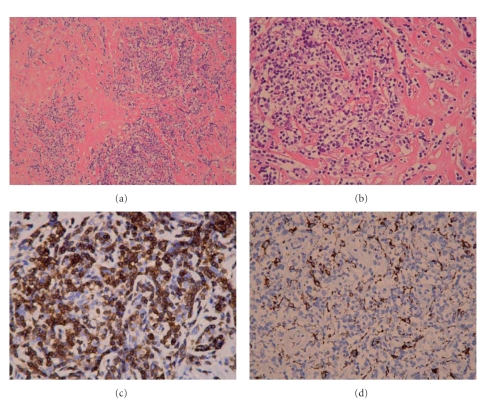
Low (a) and high (b) magnifications of hematoxylin-eosin-safron stained sections showing nodular cellular infiltrates with abundant fibrosis; immunohistochemistry revealing a prevalence of CD3-positive T lymphocytes (c) and some CD68-positive histiocytes (d).

The patient was treated with diuretics, angiotensin-converting enzyme inhibitors, spironolactone, amiodarone, and adequate anticoagulation (INR 2.0-3.0) with warfarin associated with steroid therapy (prednisolone 1 mg/kg/day). Right ventricular myocardial biopsy and implantation of a dual-chamber epicardial pacemaker were performed via a sternal thoracotomy. Hematoxylin-eosin-saffron stained sections of the tissue sample showed a granulomatous reaction consisting of nodular cellular infiltrates (histiocytes associated with lymphocytic elements) with an abundant fibrotic reaction (Figures 5(a) and 5(b)). Immunohistochemistry revealed a prevalence of T lymphocytes (CD3 positive; Figure 5(c)) mixed with B lymphocytes (CD20 positive), histiocytes (CD68 positive; Figure 5(d)) and a few plasma cells (CD138 positive). Neither giant cells nor epithelioid cells were observed, thus rejecting the diagnoses of giant cell myocarditis and cardiac sarcoidosis. Vessels were not specifically concerned with the cellular infiltrates, so that a diagnosis of vasculitis was also excluded. Congo red and hematoxylin-eosin stained sections did not show amyloid deposits. Nested/reverse transcription-PCR did not detect any genomes of enterovirus, adenovirus, parvovirus B19, or human herpes virus type 6.

At followup twelve months later, the patient described NYHA class II dyspnea, with no clinical signs of heart failure. Echocardiography revealed persistent myocardial infiltration, although the pericardial effusion had resolved. The patient remained pacemaker dependent with underlying complete heart block, which is probably the consequence of infiltration of the cardiac conduction system with granulomatous tissue. Nt-proBNP had decreased to 550 pg/mL.

## 3. Discussion

We report a fairly special case of lymphocytic myocarditis progressing to a fibrotic stage, described using multi-modality imaging and confirmed on histopathology. This paper presents an uncommon diagnosis with a probable guarded prognosis.

First, although MRI and other imaging modalities findings are relatively nonspecific, we can notice the ability of dark-blood T2-weighted and first-pass perfusion imaging to detect, respectively, oedema and enhancement of the right ventricular free wall and the interventricular septum infiltration consistent with an active inflammatory process. Consequently, we decided a corticosteroid therapy in order to clear inflammation. Furthermore, we confirm in this paper CMR's ability to depict myocardial fibrosis on delayed enhancement imaging. We can suppose that this fibrous infiltration of the right ventricle and basal to mid interventricular septum is responsible for the persistence of conduction disturbances. Thus, cardiac magnetic resonance imaging detected oedema, residual inflammatory process, and myocardial fibrosis, which may influence patient's management. We regret that a second CMR control could not be performed because of the pacemaker. 

Secondly, this patient presented incidentally with apparently idiopathic cardiac granulomatosis, probably being longstanding. We used the term “granulomatous” because of the nature of nodular lesions and the presence of histiocytes associated with lymphocytic elements. Cardiac sarcoidosis and idiopathic giant cell myocarditis are usually clinicopathologic entities evocated in this situation. Okura et al. [2] demonstrated, in a large series of patients, histological differences between cardiac sarcoidosis and idiopathic giant cell myocarditis: the first has more granulomas and fibrosis, while the second one more necrosis, foci of lymphocytic myocarditis and eosinophils. The number of giant cells is equivalent in the two entities. Presentation with heart failure predicted idiopathic giant cell myocarditis, whereas presentation with heart block or more than nine weeks of symptoms is associated with cardiac sarcoidosis. There is still an open debate as to whether idiopathic giant cell myocarditis and cardiac sarcoidosis are separate disorders. However, in this case, neither giant cells nor epithelioid cells were observed, thus rejecting the diagnoses of giant cell myocarditis and cardiac sarcoidosis. Thus, a viral-negative lymphocytic myocarditis which had progressed to a fibrotic and granulomatous stage was the most plausible diagnosis, although we recognize that infiltration of the aortic root and the right atrium is very unusual in case of myocarditis. Surprisingly, in this patient presenting with chronic myocarditis and persistent inflammation, nested/reverse transcription-PCR did no detect any genomes of virus. Gutberlet et al. [3] observed that viral genomes were amplified at nested PCR assay in 49 (59%) of the 83 patients presenting with chronic myocarditis. Parvovirus B19 was the most frequently detected virus. Because lymphocytic myocarditis has multiple etiologies, therapy should be individualized according to the specific etiology. Unfortunately, efficacy of most therapies used or proposed for use in lymphocytic myocarditis has not been proven. Until this proof is available, specific and nonspecific antiviral measures should be considered for use in those patients who present with documented ongoing viral infection. Likewise, appropriate antimicrobial is always indicated in lymphocytic myocarditis caused by bacterial and other organisms. The area of greatest controversy is the use of immunosuppressive and other immunomodulatory therapies for noninfectious and postinfectious myocarditis. In our case we hypothesize that corticosteroid therapy may be effective in clearing the residual inflammatory process. Healing of myocarditis lesions may result in some degrees of fibrosis [4–6]. Earlier series have shown that symptoms do not reflect the extent of lymphocytic infiltrate and fibrosis [7]. However, we can notice that our patient presented with severe heart failure, and in parallel the endomyocardial biopsy showed significant fibrosis. 

Thirdly, this paper presents an uncommon diagnosis with a probable guarded prognosis. Magnani et al. [8] reported in 112 patients with histopathologic confirmation of myocarditis that 88 (79%) and 63 (56%) were alive without cardiac transplantation at 1 and 5 years, respectively. Furthermore, Davidoff et al. [5] observed that the presence of atrioventricular block requiring pacemaker insertion was predictive of a subsequent fatal outcome in both giant cell and lymphocytic myocarditis. 

## 4. Conclusion

We report a fairly special case of lymphocytic myocarditis progressing to a fibrotic stage, described using multi-modality imaging and confirmed on histopathology. The combination of CMR with various image acquisition sequences associated with other imaging modalities provides a noninvasive morphological assessment and tissue characterization of myocardial involvement. This paper presents an uncommon diagnosis with a probable guarded prognosis.
